# Childhood diagnoses in individuals identified as autistics in adulthood

**DOI:** 10.1186/s13229-021-00478-y

**Published:** 2021-12-13

**Authors:** Eya-Mist Rødgaard, Kristian Jensen, Kamilla Woznica Miskowiak, Laurent Mottron

**Affiliations:** 1 Department of Psychology, University of Copenhagen, 1353 Copenhagen K, Denmark; 2 Department of Psychiatry and Addictology, Université de Montreal, Montreal, QC H3T 1J4 Canada; 3 Psychiatric Centre Copenhagen, Rigshospitalet, 2100 Copenhagen Ø, Denmark; 4 Centre de Recherche du CIUSSS-NIM, Hôpital Rivière-des-Prairies, Montreal, QC H1E 1A4 Canada

**Keywords:** Autism, Adult, Comorbidity, Late diagnosis

## Abstract

**Background:**

Autism is a developmental condition, where symptoms are expected to occur in childhood, but a significant number of individuals are diagnosed with autism for the first time in adulthood. Here, we examine diagnoses given in childhood among individuals that are diagnosed with autism in adulthood, to investigate whether the late autism diagnosis might be explained by misdiagnosis in childhood or diagnostic overshadowing.

**Methods:**

Through the Danish National Patient Registry, we identified individuals diagnosed with autism in adulthood (*N* = 2199), as well as a control sample with no records of an autism diagnosis (*N* = 460,798) and calculated how many had received different psychiatric or neurological diagnoses in childhood.

**Results:**

We found that most childhood diagnoses were overrepresented in those with an adult autism diagnosis, and attention-deficit hyperactivity disorder, affective disorders, anxiety, and stress disorders were the most prevalent childhood conditions in this group. However, 69% of males and 61% of females with adult autism diagnoses were not found to have received any of the investigated diagnoses before 18 years of age, and most childhood diagnoses were given after the age of 12.

**Limitations:**

Milder to moderate cases of psychiatric conditions that have been solely treated by family physicians or school psychologists may not be fully included in our dataset. The study is based on data from the Danish health care system, and further research is needed to assess whether the findings can be generalized to other countries.

**Conclusion:**

A majority of those with an adult autism diagnosis had no records of having received any of the investigated diagnoses in childhood. In these cases, the late autism diagnosis is therefore unlikely to be explained by either misdiagnosis or overshadowing. This result is at odds with the prevailing notion that autistic symptoms tend to diminish with age. Therefore, further research is warranted to examine how and if early signs of autism may have manifested among these individuals, and how similar they are to autistic people diagnosed earlier in their development.

**Supplementary Information:**

The online version contains supplementary material available at 10.1186/s13229-021-00478-y.

## Introduction

According to diagnostic criteria [1], symptoms of autism must appear in childhood, but there is also an increasing number of individuals who are diagnosed with autism for the first time during adulthood [2]. This trend may be part of an increase in autism heterogeneity, as individuals with less severe symptoms and lower similarity to the original descriptions of autism are also identified as autistic [3, 4]. Conversely, it has also led researchers to hypothesize a “lost generation” of autistic individuals whose autism symptoms were simply not identified in childhood [5]. It is relevant to question why the autism condition, which should have manifested across contexts during childhood, might have been missed until adulthood. It has been suggested that factors such as high IQ, female gender, and camouflaging behaviours may hinder an early identification of the condition [6, 7]. Furthermore, autism assessments in adults are associated with several challenges, e.g. less reliable information about early development and symptom overlap with other psychiatric conditions that may better explain the symptoms or co-occur with autism [5]. Some adult autism diagnoses could therefore reflect misdiagnosis due to difficulty differentiating autism symptoms from social and communicative deviances related to other psychiatric conditions such as schizophrenia [8, 9] or personality disorders [10].

Studies of autism diagnosed in adulthood generally report a high frequency of comorbid psychiatric diagnoses [11–15]. Another hypothesis therefore is that autism symptoms in childhood were mistaken as symptoms of other conditions [14, 16] such as attention-deficit hyperactivity disorder (ADHD) [17, 18] or obsessive–compulsive disorder (OCD) [19] due to symptom overlap. It is also possible that both conditions were present, but due to diagnostic overshadowing, the autism symptoms were attributed to the other condition and no autism diagnosis was given [20, 21].

Although numerous studies have investigated previous psychiatric diagnoses among individuals diagnosed with autism as adults, most of these studies did not investigate *when* previous diagnoses were given, i.e. in childhood or relatively shortly before the autism diagnoses well into adulthood. This is important in order to differentiate late autism diagnoses caused by misdiagnosis or overshadowing in childhood, from those reflecting a late emergence of any noticeable symptoms. Here, we specifically investigate childhood deviation in individuals who were diagnosed with autism in adulthood. We used a Danish national health registry to investigate which diagnoses these individuals were given in childhood, and thus whether misdiagnosis and diagnostic overshadowing might explain why autism can have gone undiagnosed throughout childhood.

## Method

**Fig. 1:**
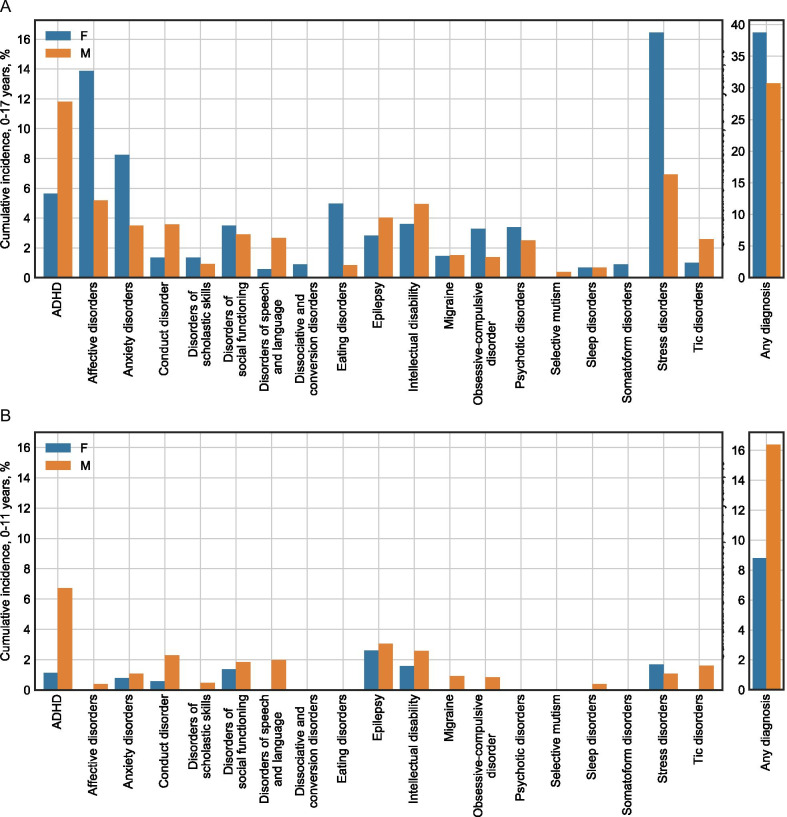
Childhood prevalence of diagnoses among males and females diagnosed with autism as adults. **a** Diagnoses given before the age of 18. **b** Diagnoses given before the age of 12

Data were retrieved from the Danish National Patient Registry (DNPR), which contains information about all diagnoses given within the Danish hospital sector (in-patient and out-patient) from 1994 to 2018. The DNPR is connected to the Danish Central Person Registry, allowing diagnoses given to the same individual at different times to be linked. Access to the registries was obtained through the Danish Health Data Authority. To ensure that the available data covered all relevant diagnoses given in childhood, we excluded individuals born before 1 January 1993. Our autism population thus consisted of individuals born between 1 January 1993 and 31 December 1999 who were diagnosed with autism (ICD-10 codes F84.0, F84.1, F84.5, F84.8, F84.9) at age 18 or later (1312 males, 887 females). As a control population, we included all individuals from the same birth years, who were not registered as having received an autism diagnosis. In each group, we calculated the percentage of males and females that had received one of the psychiatric or neurological diagnoses shown in Fig. 1 before the age of 18, and the median age at diagnosis. For each diagnosis, odds ratios (OR) were calculated to compare males and females, the diagnosis groups (adult autism diagnosis compared to no autism diagnosis), and the interaction between the two. Statistical significance of OR’s was calculated by fitting binomial regression models using the “glm” function in R version 3.6.2 and performing analysis of deviance using the “ANOVA” function of the “car” package version 3.0-6 [22]. The ICD-10 codes used for each diagnosis are shown in Additional file 1: Table S2. The diagnoses were chosen based on what previous studies have found in populations diagnosed with autism in adulthood, as well as diagnoses as which autism might be misclassified due to symptom overlaps. The data were extracted on 24 August 2020.

## Results

The childhood prevalence (0–17 years of age) of each selected diagnosis among those diagnosed with autism in adulthood is shown in Fig. 1a and Table 1. All the investigated childhood diagnoses, except migraine and sleep disorder, were found significantly more often among those diagnosed with autism in adulthood, compared to those who had never received an autism diagnosis (Table 1). For all childhood diagnoses except disorders of scholastic skills, no interaction effect between sex and adult autism diagnosis was found, indicating that an adult autism diagnosis was associated with the same increase in risk for childhood diagnosis in males and females. Despite an overrepresentation of childhood diagnoses, 69% of males and 61% of females with an adult autism diagnosis had not been registered with any of the selected diagnoses before their 18th birthday. Furthermore, the median age of receiving childhood diagnoses was greater than 10 years for most of the diagnoses (Table 1), and only 16% of males and 9% of females had received any of the diagnoses before the age of 12 (Fig. 1b).

**Table 1:** Percentages of childhood diagnoses (from 0 to 17 years of age) among Danish individuals born between 1993 and 1999

	Autism diagnosed in adulthood			No autism diagnosis			Comparisons		
	Females *N* = 887 %	Males *N* = 1312 %	Median age Females/males (years)	Females *N* = 226,870 %	Males *N* = 233,928 %	Median age Females/males (years)	OR (adult autism)	OR (gender)	Interaction ΔlogOR
ADHD	5.6	11.8	15/11	1.7	3.5	14/11	3.52***	0.46***	− 0.03
Affective disorders	13.9	5.2	16/16	2.7	0.9	16/15	5.80***	2.91***	0.01
Anxiety disorders	8.2	3.5	16/14	1.8	1.0	15/13	4.88***	1.76***	0.33
Conduct disorder	1.4	3.6	12.5/10	0.3	0.7	13/10	4.66***	0.41***	− 0.11
Disorders of scholastic skills	1.4	0.9	15/11.5	0.2	0.4	15/12	5.49***	0.63***	0.86*
Disorders of social functioning	3.5	2.9	13/10	0.4	0.6	12/9	8.30***	0.78***	0.44
Disorders of speech and language	0.6	2.7	12/7	0.2	0.4	7/6	2.99***	0.43***	− 0.72
Dissociative and conversion disorders	0.9	< 0.4	15.5/NA	0.2	0.0	15/15	5.68**	4.41***	NA
Eating disorders	5.0	0.8	15/13	1.7	0.2	15/12	3.10***	7.84***	− 0.24
Epilepsy	2.8	4.0	4/5	1.7	1.7	7/7	1.71***	0.98	− 0.35
Intellectual disability	3.6	5.0	13/11	0.8	1.0	12/10	4.89***	0.73***	− 0.01
Migraine	1.5	1.5	15/10.5	1.2	1.0	13/12	1.25	1.22***	− 0.24
Obsessive–compulsive disorder	3.3	1.4	15/10	0.7	0.5	14/13	4.48***	1.51***	0.48
Psychotic disorders	3.4	2.5	16/15	0.7	0.4	15/15	5.02***	1.65***	− 0.20
Selective mutism	< 0.6	0.4	NA/7	0.0	0.0	7/10	31.96***	2.21**	NA
Sleep disorders	0.7	0.7	16.5/8	0.5	0.6	9/6	1.42	0.86***	0.13
Somatoform disorders	0.9	< 0.4	14/NA	0.3	0.1	14/14	2.99*	3.28***	NA
Stress disorders	16.5	6.9	15/15	4.7	2.0	15/14	4.00***	2.37***	0.11
Tic disorders	1.0	2.6	15/11	0.3	0.8	11/10	4.01***	0.30***	0.23
Any of the above	38.8	30.7		12.4	10.5		4.50***	1.20***	0.17

The first three columns show childhood diagnoses among those diagnosed with autism in adulthood and the median ages at diagnosis. The middle three columns show the same information for those with no record of an autism diagnosis. The last three columns show comparisons of childhood diagnosis between those with and without an adult autism diagnosis and between females and males. Odds ratios (OR) of 1 correspond to null effects, while an OR larger than 1 indicate a higher prevalence of childhood diagnosis in females or those with an adult autism diagnosis, respectively. For the interaction effect, a Δlog(OR) of 0 corresponds to a null effect, while positive values indicate that the sex-related OR is higher in the adult autism group. Significance levels: * = *p* < 0.05, ** = *p* < 0.001, *** = *p* < 0.0001

## Discussion

The presence of childhood diagnoses of psychiatric or neurological disorders may explain why autism has not been diagnosed throughout childhood, e.g. due to misdiagnosis or diagnostic overshadowing. The present data showed that although childhood diagnoses were significantly overrepresented among individuals with an adult autism diagnosis, only 31% of males and 39% of females were registered as having received any of the diagnoses shown in Fig. 1 before the age of 18, and even fewer were registered with any of the diagnoses before the age of 12 (16% of males and 9% of females).

We found high incidences of ADHD, mood disorders, and anxiety before the age of 18, which is consistent with previous studies of comorbidity in individuals diagnosed with autism in adulthood [11–15, 23]. Incidences before the age of 12 were considerably lower, showing that most of the pre-autism childhood diagnoses were given in adolescence. Sex differences of childhood diagnoses in those with an adult autism diagnosis were largely not significantly different from those in the general population. There was a high incidence of stress disorders before the age of 18, particularly among females, whereas previous studies have either not reported rates of stress disorders [11, 14] or only reported rates specifically for post-traumatic stress disorder [12, 13, 15]. The fraction of individuals who had received any of the investigated diagnoses in childhood was slightly lower than what has previously been reported. Rydén and Bejerot [13] found that 53% of those diagnosed with autism as adults had received psychiatric care in childhood, while Geurts and Jansen [14] found that 53% had been in contact with the mental health system in childhood, and 32% and 51% had received a psychiatric diagnosis of axis I or axis II disorders, respectively, prior to their adult autism diagnosis. The variations might be attributed to changes in the autism population over time [24], or differences in which diagnoses were included. It is also possible that the difference can be ascribed to the fact that the DNPR only contain diagnoses given in the hospital system, while some individuals may have received childhood psychiatric care in the primary healthcare sector, e.g. for conditions such as depression or anxiety [25].

In cases with childhood diagnoses, the delayed autism diagnosis may be attributed to misdiagnosis or overshadowing. Similarly, there may have been a reclassification of diagnoses, such that symptoms that are now identified as autistic would previously have been attributed to another condition, e.g. intellectual disability [26]. However, the large remaining majority of late autism diagnoses likely have other explanations. Some individuals may not have exhibited signs of autism to a degree that has caused a thorough diagnostic evaluation to be initiated. Recent evidence suggests that the threshold for receiving an autism diagnosis has been lowered through the last decades [4], which may also explain why a person that did not meet the criteria in childhood can get a diagnosis later in life. Furthermore, in some cases symptoms may have been missed either in the mental health system or by the systems that should have referred a child for assessment. Although health care is free in Denmark, this may particularly affect individuals of lower socioeconomic status, whose parents may not have the resources to successfully pursue support for their children.

### Camouflaging early symptoms

Some researchers have hypothesized the concept of camouflaging, describing coping strategies used to hide autism symptoms and maintain an appearance of normal social functioning [7]. Studies of camouflaging have suggested that the coping behaviour requires an intense effort [7], which may eventually cause distress to a degree where a psychiatric evaluation is initiated, leading to a late autism diagnosis [27]. There have been concerns about the validity of the camouflaging construct and suggestions that further research is needed to operationalize it and differentiate it from other constructs [28]. For instance, the differentiation between camouflaging and the normal learning of socially accepted behaviour, which occurs in the non-autism population, is not clearly established. This distinction is of particular importance in regard to proposed unconscious camouflaging behaviour (e.g. [7, 29]). Additionally, in order for camouflaging to result in autism being missed, the camouflaging behaviour would need to begin very shortly after the onset of symptoms, which would require a child to quickly grasp complex elements of the social world at an early age and understand them well enough to pass as non-autistic. Although this could in theory explain our results of absence of childhood diagnoses, such adept navigation of social contexts contrasts somewhat with the deficits of social understanding and interaction that have traditionally been a core concept of autism [30, 31]. Consequently, a history of successful camouflaging of supposed autism symptoms might be considered as proof of the absence of clinically relevant autism symptoms to begin with. Thus, it is necessary to strengthen the validity of the distinction between camouflaged symptoms and absence of symptoms, in order to elucidate to which extent camouflaging contributes to individuals with an adult autism diagnosis not being diagnosed in childhood.

### Autism with late emergence

Another explanation for adult autism diagnoses with no apparent psychiatric problems in childhood is that some individuals simply develop autism symptoms later than in the traditional developmental trajectory of autism. In a large general population longitudinal cohort, a subgroup consisting of 7.3% of the cohort was found to have autistic traits that increased over time, particularly during the 10–16-year age period [32]. This could suggest that these individuals develop a late-onset variant of autism. However, before one concludes that some individuals have a late onset of autism, it is necessary to consider an alternative hypothesis, namely that these individuals have another condition that may share elements with autism but is not identical to autism. In other words, their symptoms might have a resemblance with autism, but the aetiology and neurological underpinnings may well be different. Studies finding evidence of late-onset autistic traits [32, 33] are generally based on instruments such as the social and communication disorders checklist (SCDC) that measure “autistic social traits”, which do not cover all aspects of autism symptoms. Although they measure traits that have been found to be elevated in individuals with autism, the inverse claim, i.e. that individuals with high scores for autistic traits are necessarily autistic, is logically wrong. Items in autistic traits instruments may be endorsed by people with a variety of psychiatric conditions for a variety of reasons [34], and the specificity of SCDC for discriminating between autism and other psychiatric conditions has indeed been found to be low [35]. Consequently, an increase in SCDC scores during adolescence might indicate development of other psychiatric conditions or a response to stressors such as increased social and executive requirements during the transition into adolescence or adulthood. It is thus currently unclear whether insights into autism can be gained by studying “autistic social traits” in populations without an actual autism diagnosis [34, 36, 37].

It is an open question whether cases with a late onset of symptoms associated with autism should be grouped together with more typical cases of autism, by amending the diagnostic criteria to allow diverse trajectories, or whether these cases should be considered separately. This issue is not unique to the field of autism. Recently, a large number of cases with late-onset tic disorders have been observed across different countries [38, 39]. Despite some resemblance to Tourette’s syndrome (TS), these tics have generally been categorized separately, e.g. as functional tic-like behaviours (FTLB) [39]. Research has shown differences between TS tics and FTLB’s, pertaining to pathophysiology and response to treatment [40]. Similar comparative studies of childhood autism and late-onset autistic traits would be beneficial in order to elucidate scientifically and clinically relevant commonalities and differences between them [41].

## Limitations

The present results are based on records of autism diagnoses and cannot inform on the correspondence to a “true” autism condition. Because our data only contains diagnoses given within the hospital sector, some individuals with an adult autism diagnosis may not be included if they were assessed and treated exclusively by their primary physician or a psychiatrist in private practice. However, since diagnostic assessment for autism in adults is complicated by several factors [5], diagnoses not conducted by specialist teams might have insufficient validity and include false-positives. The data also do not include childhood diagnoses given by school psychologists or primary physicians. However, problems that are managed solely by a primary physician or school psychologist (e.g. milder cases of anxiety or depression) are likely less pronounced than the cross-context challenges that would be expected due to autism. Finally, the results are based only on data from a Danish population, and replication in other populations is necessary to establish the generalizability of the findings to other countries.

## Conclusion

Several explanations may have contributed to the delayed autism diagnoses of the individuals in the current study. Most individuals receiving an autism diagnosis in adulthood did not receive any of the investigated psychiatric diagnoses during childhood. In such cases, the late autism diagnosis cannot likely be explained by misdiagnosis or overshadowing. Rather, an adult autism diagnosis could possibly be caused by camouflaging behaviour or atypical trajectories. Further research is warranted to investigate how autism may have manifested in childhood in these individuals, whether they might have benefited from access to support, and whether cases of autism that only reach a clinical threshold in adulthood are developmentally, phenomenologically, and biologically distinct from their childhood counterparts. Future research and discussion within the field should address whether cases with late emergence of symptoms should be classified with a diagnosis other than autism, or whether the definition of autism should be adapted to allow for such atypical developmental trajectories.

## Supplementary Information

**Additional file 1.** Supplementary tables and figures.

## Data Availability

The aggregated count data described in this manuscript are included in the supplementary material. The raw data in the Danish National Patient Registry cannot be shared. Researchers can apply for access to the raw data through the Danish Health Data Authority.

## Abbreviations

ADHD: Attention-deficit hyperactivity disorder
DNPR: Danish National Patient Registry
FTLB: Functional tic-like behaviour
OCD: Obsessive compulsive disorder
OR: Odds ratio
SCDC: Social and communication disorders checklist
