# Supplementary material for: Childhood diagnoses in individuals identified as autistics in adulthood
Source: Mol Autism. 2021 Dec 13;12:73. doi: 10.1186/s13229-021-00478-y (PMC8670291; doi:10.1186/s13229-021-00478-y)
Supplement: Supplementary file 1 — Additional file 1. Supplementary tables and figures. [file 13229_2021_478_MOESM1_ESM.docx]

Table S1: Percentages of individuals with childhood diagnoses (from 0-11 years of age) among Danish individuals born between 1993 and 1999. The first two columns show childhood diagnoses among those diagnosed with autism in adulthood, and the last two columns show childhood diagnoses among those with no record of an autism diagnosis. In groups where less than 5 individuals received a given diagnosis, the exact percentage is not shown.

|  | Autism diagnosed in adulthood | |  | |
| --- | --- | --- | --- | --- |
|  |  |  | *No autism diagnosis* | |
|  | **Females** | **Males** | **Females** | **Males** |
|  | **N=** **887** | **N=** **1,312** | **N=** **226,870** | **N=** **233,928** |
|  | **%** | **%** | **%** | **%** |
| **ADHD** | 1.13 | 6.71 | 0.45 | 1.85 |
| **Affective disorders** | <0.45 | 0.38 | 0.06 | 0.10 |
| **Anxiety disorders** | 0.79 | 1.07 | 0.27 | 0.39 |
| **Conduct disorder** | 0.56 | 2.29 | 0.11 | 0.42 |
| **Disorders of scholastic skills** | <0.45 | 0.46 | 0.06 | 0.17 |
| **Disorders of social functioning** | 1.35 | 1.83 | 0.20 | 0.36 |
| **Disorders of speech and language** | <0.45 | 1.98 | 0.12 | 0.36 |
| **Dissociative and conversion disorders** | <0.45 | <0.30 | 0.01 | 0.01 |
| **Eating disorders** | <0.45 | <0.30 | 0.18 | 0.10 |
| **Epilepsy** | 2.59 | 3.05 | 1.19 | 1.31 |
| **Intellectual disability** | 1.58 | 2.59 | 0.36 | 0.60 |
| **Migraine** | <0.45 | 0.91 | 0.40 | 0.48 |
| **Obsessive-compulsive disorder** | <0.45 | 0.84 | 0.16 | 0.18 |
| **Psychotic disorders** | <0.45 | <0.30 | 0.02 | 0.04 |
| **Selective mutism** | <0.45 | <0.30 | 0.02 | 0.01 |
| **Sleep disorders** | <0.45 | 0.38 | 0.27 | 0.39 |
| **Somatoform disorders** | <0.45 | <0.30 | 0.05 | 0.03 |
| **Stress disorders** | 1.69 | 1.07 | 0.48 | 0.57 |
| **Tic disorders** | <0.45 | 1.60 | 0.14 | 0.55 |
| **Any of the above** | 8.79 | 16.39 | 3.59 | 5.79 |

Table S2: Diagnostic codes for each of the investigated conditions. Presence of a condition was defined as having a diagnosis with at least one of the codes (or a subcode) for a given condition.

|  | **ICD-10 codes** |
| --- | --- |
| **ADHD** | F90 |
| **Affective disorders** | F30, F31, F32, F33, F34 |
| **Anxiety disorders** | F93, F40, F41 |
| **Conduct disorder** | F91 |
| **Disorders of scholastic skills** | F81 |
| **Disorders of social functioning** | F94 |
| **Disorders of speech and language** | F80 |
| **Dissociative and conversion disorders** | F44 |
| **Eating disorders** | F50 |
| **Epilepsy** | G40 |
| **Intellectual disability** | F70-F79 |
| **Migraine** | G43 |
| **Obsessive-compulsive disorder** | F42 |
| **Psychotic disorders** | F20-F29 |
| **Selective mutism** | F94.0 |
| **Sleep disorders** | F51, G47 |
| **Somatoform disorders** | F45 |
| **Stress disorders** | F43 |
| **Tic disorders** | F95 |

Table S3: Percentages of males and females with adult autism diagnoses, who were given each childhood diagnosis in different age brackets. Each individual is only counted in the age bracket where they first received the given diagnosis. Blank cells indicate that less than 5 individuals received a given diagnosis in a given age brackets. For females this corresponds to less than or equal to 0.45%, while for males it corresponds to less than or equal to 0.30%.

|  | **Female (N = 887)** | | | **Male (N = 1312)** | | |
| --- | --- | --- | --- | --- | --- | --- |
| **Age at first diagnosis (years)** | **0-5** | **6-11** | **12-16** | **0-5** | **6-11** | **12-17** |
|  |  |  |  |  |  |  |
| **ADHD** |  | 1.13 | 4.51 | 1.3 | 5.41 | 5.11 |
| **Affective disorders** |  |  | 13.64 |  | 0.38 | 4.8 |
| **Anxiety disorders** |  | 0.68 | 7.44 |  | 0.99 | 2.44 |
| **Conduct disorder** |  |  | 0.79 | 0.38 | 1.91 | 1.3 |
| **Disorders of scholastic skills** |  |  | 1.13 |  | 0.46 | 0.46 |
| **Disorders of social functioning** | 0.9 |  | 2.14 | 0.46 | 1.37 | 1.07 |
| **Disorders of speech and language** |  |  |  | 1.22 | 0.76 | 0.69 |
| **Dissociative and conversion disorders** |  |  | 0.9 |  |  |  |
| **Eating disorders** |  |  | 4.74 |  |  | 0.61 |
| **Epilepsy** | 1.8 | 0.79 |  | 2.06 | 0.99 | 0.99 |
| **Intellectual disability** | 0.68 | 0.9 | 2.03 | 1.07 | 1.52 | 2.36 |
| **Migraine** |  |  | 1.24 |  | 0.76 | 0.61 |
| **Obsessive-compulsive disorder** |  |  | 2.82 |  | 0.76 | 0.53 |
| **Psychotic disorders** |  |  | 3.27 |  |  | 2.21 |
| **Sleep disorders** |  |  | 0.56 |  |  |  |
| **Somatoform disorders** |  |  | 0.56 |  |  |  |
| **Stress disorders** |  | 1.47 | 14.77 |  | 0.76 | 5.87 |
| **Tic disorders** |  |  | 0.56 |  | 1.37 | 0.99 |

Table S4: Raw count data used for statistical analyses. Shows how many individuals with and without an autism adult diagnosis received each childhood diagnosis (0-17 years).

|  | **No autism** | | **Adult autism diagnosis** | |
| --- | --- | --- | --- | --- |
| **Sex** | **Female** | **Male** | **Female** | **Male** |
| **N** | **226870** | **233928** | **887** | **1312** |
|  |  |  |  |  |
| **ADHD** | 3781 | 8287 | 50 | 155 |
| **Affective disorders** | 6123 | 2208 | 123 | 68 |
| **Anxiety disorders** | 4098 | 2418 | 73 | 46 |
| **Conduct disorder** | 666 | 1662 | 12 | 47 |
| **Disorders of scholastic skills** | 565 | 926 | 12 | 12 |
| **Disorders of social functioning** | 986 | 1297 | 31 | 38 |
| **Disorders of speech and language** | 429 | 1033 | 5 | 35 |
| **Dissociative and conversion disorders** | 363 | 85 | 8 | <5 |
| **Eating disorders** | 3762 | 502 | 44 | 11 |
| **Epilepsy** | 3782 | 3986 | 25 | 53 |
| **Intellectual disability** | 1722 | 2440 | 32 | 65 |
| **Migraine** | 2664 | 2257 | 13 | 20 |
| **Obsessive-compulsive disorder** | 1698 | 1163 | 29 | 18 |
| **Psychotic disorders** | 1570 | 981 | 30 | 33 |
| **Selective mutism** | 60 | 28 | <5 | 5 |
| **Sleep disorders** | 1084 | 1294 | 6 | 9 |
| **Somatoform disorders** | 689 | 217 | 8 | <5 |
| **Stress disorders** | 10656 | 4762 | 146 | 91 |
| **Tic disorders** | 579 | 1944 | 9 | 34 |
| **Any of the above** | 28050 | 24578 | 344 | 403 |

Figure S1: Visualization of the data in Table S3 showing when individuals with an adult diagnosis of autism first received a childhood diagnosis. The bars indicate how large a percentage of males (top) and females (bottom) first received a given diagnosis in the age brackets of 0-5 years, 6-11 years and 12-17 years of age.
